# The effects of publishing emergency department wait time on patient utilization patterns in a community with two emergency department sites: a retrospective, quasi-experiment design

**DOI:** 10.1186/1865-1380-4-29

**Published:** 2011-06-14

**Authors:** Bin Xie, Sabrina Youash

**Affiliations:** 1 Department of Epidemiology & Biostatistics, Schulich School of Medicine and Dentistry, University of Western Ontario, London, ON, Canada; 2 Department of Obstetrics and Gynecology, Schulich School of Medicine and Dentistry, University of Western Ontario, London, ON, Canada; 3 Room E5-319, LHSC-VH, 800 Commissioners Road East, London, Ontario, N6A 5W9, Canada

## Abstract

**Background:**

Providing emergency department (ED) wait time information to the public has been suggested as a mechanism to reduce lengthy ED wait times (by enabling patients to select the ED site with shorter wait time), but the effects of such a program have not been evaluated. We evaluated the effects of such a program in a community with two ED sites.

**Methods:**

Descriptive statistics for wait times of the two sites before and after the publication of wait time information were used to evaluate the effects of the publication of wait time information on wait times. Multivariate logistical regression was used to test whether or not individual patients used published wait time to decide which site to visit.

**Results:**

We found that the rates of wait times exceeding 4 h, and the 95th percentile of wait times in the two sites decreased after the publication of wait time information, even though the average wait times experienced a slight increase. We also found that after controlling for other factors, the site with shorter wait time had a higher likelihood of being selected after the publication of wait time information, but there was no such relationship before the publication.

**Conclusions:**

These findings were consistent with the hypothesis that the publication of wait time information leads to patients selecting the site with shorter wait time. While publishing ED wait time information did not improve average wait time, it reduced the rates of lengthy wait times.

## Background

Lengthy wait time for access to healthcare services is a persistent issue in many jurisdictions [1,2], and to make matters worse, wait times for different providers in the same area can vary significantly [3,4]. While solutions to these problems usually involve capacity increases or efficiency improvement, with the adoption of information technologies in the healthcare system, an alternative or supplementary approach has been proposed and implemented in some jurisdictions: publishing wait time information of different providers so that patients can make informed choices on which provider to use [5-8].

There are good reasons to believe that such an approach might help reduce lengthy wait times. For example, patients are known to be willing to travel or to switch healthcare providers in order to achieve shorter wait times [9-14], and if more patients choose providers with shorter wait times, then lengthy wait times might be less likely to occur. Such effects, however, have not been empirically demonstrated.

One of the most promising areas of such application is emergency department (ED) care, as lengthy waits in EDs are widespread in many communities [15-18]. Wait times in different ED sites in the same community can differ significant [8,19,20], and patients' selection of providers is less constrained by factors such as referral, continuity of care, and familiarity with providers, as patients usually do not require a referral to visit an ED, and patients usually do not have the ability to select care providers in an ED.

In this study, we aim to test the hypothesis that publication of wait time information would lead to more patients visiting the ED site with shorter published wait time, and that such a change in utilization pattern would lead to reduction in lengthy wait times in a community with two ED sites. This study was reviewed and approved by the Research Ethics Board at the University of Western Ontario.

## Methods

### Study setting and data source

The study setting is a middle-sized community with a population of half a million with two adult ED sites in southwest Ontario, Canada. These two sites are: University Hospital (UH) and Victoria Hospital (VH). The two sites are 8.5 km apart (travel distance by road), and in normal traffic conditions it takes approximately 15 min to travel from one site to another by car, and about 30 min by public transportation. The two sites are staffed by the same group of physicians.

Starting from 19 February 2009, the average wait time information of these two sites has been available to the public (updated daily) via a website [8]. The number of "hits" to the website ranged from 18 to 39 per day during the month of June 2010 (historical data for number of "hits" per day are not available), suggesting that around 10% to 20% of the visitors were using this website.

Administrative ED records for these two hospitals were used in the data analysis. Such ED records include demographics (age, gender, postal code) for the patients and timing (time of triage, admission, initial physician assessment, and discharge) and clinical information (triage level, main reason for the visit, discharge) of the visits. We used ED records during the period from 1 August 2008 to 31 August 2009, excluding the transition period of February 2009. The period between 1 August 2008 and 31 January 2009 will be referred to as the "before" period hereafter, and the period between 1 March 2009 and 31 August 2009 the "after" period. A dummy variable, "period," was created to represent the period (0 as the "before" period and 1 "after").

Patients aged 19 and above who self-arrived at the ED (i.e., not by ambulance), whose triage levels were not "Resuscitation" or "Emergency," who provided a valid residential postal code, and who resided within 50 km from either site were included in the analysis, as these patients are the most likely to use the published wait time information to select which site to visit. Those who resided more than 50 km away were excluded as they were most likely visiting the region rather than traveling from their home to visit the ED, making their travel distances impossible to calculate. Follow-up visits were also excluded as such visits did not involve a choice of site. Independent visits from the same patient were treated as separate visits as the same patient may visit different sites at different times. Please note that ability to pay was not a factor because of Canada's universal healthcare system.

### Measurements

Patient age was grouped into three categories: 19-40, 41-60, and 61 and older. Age was not treated as a continuous variable as we believe that age is likely to have a non-linear effect [20]. We chose 40 and 60 as the two cutoff points as we believed that people below and above these two ages may have important differences in their reactions to information on the Internet. Travel distances between the patients' resident addresses and the two ED sites were calculated using ArcGIS 9.3.1, a Geographical Information Systems (GIS) software product from Environmental Systems Research Institute, Inc. (ESRI). The latest Ontario road map files were used, with longitude and latitude values of the patients' postal codes obtained from Statistics Canada Post Code Conversion File (PCCF) [21]. The accuracy of PCCF in measuring distances between two postal codes was validated elsewhere [22]. Differences in travel distances between the patient's residence and the two ED sites were grouped into two categories: UH closer and VH closer. Another patient level variable, gender, was also included in the model.

Time of the day was grouped into three categories based on triage time: daytime (8 a.m.-4 p.m.), early evening (4 p.m.-12 a.m.), and midnight (12 a.m.-8 a.m.). Such categorization corresponds to the time shifts of the ED sites, with changes in staffing and other resources. Days of the week were grouped into two categories: weekdays (Monday through Friday) and weekends (Saturday and Sunday) based on triage time.

Main reason for visit was categorized into five categories using ICD 10 codes: "Mental and behavioral disorders" (ICD codes starting with F), "Pregnancy, childbirth, and the puerperium" (ICD codes starting with O), "Diseases of the circulatory system" (ICD codes starting with I), "Injury, poisoning, or external causes of morbidity and mortality" (ICD codes starting with S, T, V, or Y), and other reasons.

Level of emergency was determined at triage (triage level). There are five possible levels: Resuscitation, Emergency, Urgent, Less Urgent, and Non Urgent. As mentioned above, those with triage levels Resuscitation or Emergency were excluded.

Wait time was defined as the time between registration and discharge ("door to door"). Another measure of ED wait time, namely the time between registration and initial assessment by a physician ("door to doctor"), was also used in the literature; however, since such wait time was not published in the website, we did not use it in the analysis. Differences in wait times were grouped into three categories: No difference; UH shorter; VH shorter.

### Data analysis

Rate of wait time exceeding 4 h and the mean, standard deviation, and 95th percentile of wait times in the two sites were used as indicators for the likelihood of lengthy wait times in the descriptive analysis. The patients' selection of ED sites was used as the outcome variable in the logistic regression. Independent variables include the patients' demographic and clinical variables, the characteristics of the timing of the visit, and the distances between the patients' residences to the two ED sites.

Univariate analyses were performed on all covariates, and those with a *p *value less than 0.10 were included in the multivariate model. An interaction variable between the difference in wait time and the dummy variable "period" was included in the multivariate model to test the hypothesis that the publication of wait time information led to more patients selecting the site with shorter wait time. Given the likely lack of independence among the visits of the same patient, we used the Huber-White method to correct for heteroscedasticity and for correlated responses from cluster samples [23].

Data analyses were carried out using R 2.11.0 [24].

## Results

Table 1 presents the characteristics of patients in the study. There are 47,628 unique individuals with 69,687 unique visits during the study period, representing 1.46 visits per person. Of these, 34,194 had one visit, 12,099 had two to four visits, and the remaining 10,320 had five or more visits.

**Table 1:** Characteristics of study participants and their ED visits

	Aug 08-Jan 09		Mar 09-Jul 09	
	UH	VH	UH	VH
**Total number of visits**	13,134	21,464	10,785	17,740
**Time of the day**				
8 a.m. --4 p.m.	6,120 (46.6%)	9,956 (46.4%)	4,978 (46.1%)	8,037 (45.3%)
4 p.m.-midnight	4,749 (36.2%)	7,905 (36.8%)	4,040 (37.5%)	6,801 (38.3%)
Midnight-8 a.m.	2,265 (17.2%)	3,603 (16.8%)	1,767 (16.4%)	2,902 (16.4%)
**Day of the week**				
Weekdays	9,353 (71.2%)	15,423 (71.9%)	7,839 (72.7%)	12,788 (72.1%)
Weekends	3,781 (28.8%)	6,041 (28.1%)	2,946 (27.3%)	4,952 (27.9%)
**Main reason**				
Mental and behavioral disorders	242 (1.8%)	1,155 (5.4%)	196 (1.8%)	881 (5.0%)
Pregnancy, childbirth and the puerperium	121 (0.9%)	646 (3.0%)	76 (0.7%)	516 (2.9%)
Diseases of the circulatory system	550 (4.2%)	589 (2.7%)	470 (4.4%)	513 (2.9%)
Injury, poisoning, or external causes of morbidity and mortality	2,924 (22.3%)	4,971 (23.2%)	2,228 (20.7%)	4,034 (22.7%)
Other reasons	9,297 (70.8%)	14,103 (65.7%)	7,815 (72.4%)	11,796 (66.5%)
**Triage level**				
Urgent	5,269 (40.1%)	9,791 (45.6%)	4,378 (40.6%)	8,085 (45.6%)
Less urgent	7,082 (53.9%)	10,994 (51.2%)	5,923 (54.9%)	9,219 (52.0%)
Non urgent	783 (6.0%)	679 (3.2%)	484 (4.5%)	436 (2.4%)
**Wait time differences**				
UH shorter	4,665 (35.5%)	7,505 (35.0%)	4,084 (37.9%)	6,295 (35.5%)
VH shorter	6,699 (51.0%)	11,041 (51.4%)	5,907 (54.8%)	10,110 (57.0%)
Same	1,770 (13.5%)	2,918 (13.6%)	794 (7.3%)	1,335 (7.5%)
**Number of eligible patients**	10,349	16,304	8,845	14,004
**Age group**				
19-40	4,564 (44.1%)	8,054 (49.4%)	3,980 (45.0%)	7,086 (50.6%)
41-60	3,125 (30.2%)	5,185 (31.8%)	2,592 (29.3%)	4,383 (31.3%)
>61	2,660 (25.7%)	3,065 (18.8%)	2,273 (25.7%)	2,535 (18.1%)
**Gender**				
Female	5,465 (52.8%)	8,773 (53.8%)	4,679 (52.9%)	7,563 (54.0%)
Male	4,884 (47.2%)	7,531 (46.2%)	4,166 (47.1%)	6,441 (46.0%)
**Distances to two sites**				
UH closer	4,999 (48.3%)	2,755 (16.9%)	4,254 (48.1%)	2,283 (16.3%)
VH closer	5,350 (51.7%)	13,549 (83.1%)	4,591 (51.9%)	11,721 (83.7%)

Table 2 presents summary statistics of wait times in the two sites. It can be seen that the rates of wait times exceeding 4 h and the 95th percentile of the wait times in both sites had a statistically significant decrease after the intervention, even though the average wait times slightly increased in both sites (such increases did not achieve statistical significance). These results are consistent with the hypothesis that patients were using the wait time information to select which site to visit.

**Table 2:** Summary statistics of wait time and wait time differences of the two sites

Statistic	Sep 08-Jan 09	Mar 09-Jul 09	*P *value for trend
**UH rate of wait time >2 hours**	13.04%	9.81%	0.01
**VH rate of wait time >2 hours**	12.53%	9.45%	0.01
**UH wait time (mean [SD])**	106.6 [84.4]	116.3 [88.5]	0.08
**UH wait time (95% percentile)**	270.0	287.0	0.10
**VH wait time (mean [SD])**	104.7 [71.5]	112.3 [80.5]	0.15
**VH wait time (95% percentile)**	260.0	266.0	0.20

**Figure 1:**
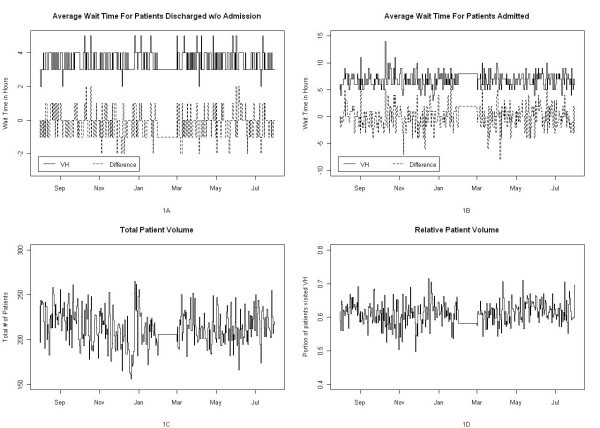
**Daily wait times and patient volumes of the two sites**.

Figure 1a presents the wait time for those discharged without admission at VH, Figure 1b presents the differences in wait times (VH-UH), Figure 1c presents the combined volume of these two sites, and Figure 1d presents the portion of patients who went to VH.

The wait times in these two sites can vary significantly, and there seemed to be no clear pattern in the relative wait times in these two sites. This variation and lack of clear pattern suggest that wait time information provides valuable information, as patients would not be able to predict the wait times of these two sites using previous experiences and/or using external factors such as time of the day or day of the week.

Table 3 presents results of the logistic regression models. As can be seen from the results, the interaction between period and wait time differences is a statistically significant predictor of patient's choice of ED site in the multivariate model, providing support for the hypothesis that patients are using the published wait time information to select which site to visit.

**Table 3:** Results of the multivariate logistical regression*

	Univariate		Multivariate	
	OR [95% CI]	*p *value	OR [95% CI]	*p *value
**Intercept**	1.64 [1.61-1.67]	<0.001	**0.67[0.62**-**0.73]**	<0.001
**Time of the day (reference: 8 a.m**.-**4 p.m.)**				
4 p.m.-midnight	1.03[1.00-1.07]	0.08	**0.94[0.91**-**0.98]**	**0.003**
Midnight-8 a.m.	1.00[0.95-1.04]	0.83	**0.94[0.89**-**0.99]**	**0.01**
**Day of the week (reference: Weekdays)**				
Weekends	1.00[0.96-1.03]	0.82	**0.98[0.94**-**1.02]**	**0.31**
**Main reason (reference: injury, poisoning, or external causes of morbidity and mortality)**				
Mental and behavioral disorders	**0.71[0.65**-**0.78]**	<0.001	**0.75[0.68**-**0.82]**	<0.001
Pregnancy, childbirth and the puerperium	**1.15[1.11**-**1.20]**	<0.001	**1.19[1.13**-**1.24]**	<0.001
Diseases of the circulatory system	**3.07[2.77**-**3.42]**	<0.001	**3.21[2.87**-**3.59]**	<0.001
Other reasons	**3.90[3.36**-**4.55]**	<0.001	**3.66[3.12**-**4.31]**	<0.001
**Triage level (reference: urgent)**				
Less urgent	**0.84[0.81**-**0.87]**	<0.001	**0.78[0.76**-**0.81]**	<0.001
Non urgent	**0.47[0.44**-**0.52]**	<0.001	**0.46[0.42**-**0.50]**	<0.001
**Wait time differences (reference: same)**				
UH shorter	0.95 [0.90-1.00]	0.07	**1.01[0.94**-**1.09]**	**0.74**
VH shorter	1.01 [0.96-1.07]	0.6	**1.02[0.95**-**1.10]**	**0.52**
**Age Group (reference: 41**-**60)**				
19-40	**0.61[0.58**-**0.63]**	<0.001	**0.60[0.57**-**0.63]**	<0.001
>61	**0.90[0.86**-**0.93]**	<0.001	**0.89[0.85**-**0.93]**	<0.001
**Gender (reference: female)**				
Male	**0.94[0.91**-**0.97]**	<0.001	**0.97[0.94**-**1.00]**	**0.08**
**Distances to two sites (reference: UH closer)**				
VH closer	**5.04[4.85**-**5.23]**	<0.001	**5.24[5.04**-**5.44]**	<0.001
**Period (reference: before)**				
After	1.01[0.97-1.04]	0.69	**1.04[0.93**-**1.17]**	**0.48**
Wait time differences * Period (reference:				
UH shorter * Period 1			**0.91[0.80**-**1.03]**	**0.14**
VH shorter * Period 1			**0.99[0.87**-**1.12]**	**0.84**

*Outcome variable: patient chose VH as the ED site

## Discussion

We found that the rates of wait times exceeding 4 h and the 95th percentile of wait times in the two sites decreased after the publication of wait time information, even though the average wait times experienced a slight increase. We also found that after controlling for other factors, the site with shorter wait time had a higher likelihood of being selected after the publication of wait time information, but there was no such relationship before the publication. These findings were consistent with the hypothesis that the publication of wait time information leads to patients selecting the site with the shorter wait time.

Due to the lack of randomization or a control community, it is difficult to establish conclusively that publication of wait time information caused patients to select the site with shorter wait time, as there may be alternative explanations of the relationship between publication of wait time information and shift in patient utilization patterns.

Nevertheless, we have reasons to believe that the results were not due to alternative explanations. More specifically, a range of factors, including local disease outbreak, construction work that made one of the ED sites more difficult to reach, or capacity changes in one or both sites, could have happened coincidently with the publication of ED wait time information and could have altered patients' selection of ED sites. However, no significant capacity change was made to either site during the study period.

Moreover, changes caused by one or a combination of these factors would persist for the entire duration during which these factors were present, but the daily volume graph did not show evidence of any sustained change.

Given the variability and the lack of clear pattern of the relative wait times of these two sites, it seems unlikely that the relative wait time would be associated with any of the aforementioned factors. Our findings provide support to the usefulness of providing wait time information to the public in addressing lengthy wait times for healthcare services.

Beyond the immediate impact on patient flows and changed wait times for those patients who use the web site, the publication of wait time information may also have broader implications.

For example, in communities with alternative providers to EDs, such as walk-in clinics or urgent care centers, publication of ED wait time information may have implications on relative patient volumes among ED sites and these alternative care providers if the patients use published ED wait times to decide not only which ED site to visit, but also whether or not to visit ED or alternative providers. This possibility is especially important in jurisdictions with universal insurance coverage such as Canada, where ability to pay is not an issue affecting patients' choices of healthcare provider. Our study does not provide any information on such implications, but it is a worthwhile topic to pursue.

Surveys also show that it is the perceived wait time, not the actual wait time, that influences patient satisfaction with EDs [25-27]. Publishing ED wait time information could enhance patient satisfaction if such published wait time is a reasonably accurate prediction of actual wait time; if the published wait time does not reflect the patient's experiences, on the other hand, patient satisfaction could be negatively affected. Given the variability in wait times of individual patients, it is unclear how accurately a summary statistic can reflect the experiences of these patients, but the implications for patient satisfaction is a topic that warrants further investigation.

## Conclusion

The findings of our study are consistent with the hypothesis that publication of wait time information would lead to more patients visiting the ED site with shorter published wait time. The impacts of such a change in utilization pattern on ED wait times were mixed: rates of wait times exceeding 4 h and the 95th percentile of the wait times in both sites had a statistically significant decrease after the publication of wait time information, although the average wait times slightly increased in both sites (such increases did not achieve statistical significance).

## Competing interests

The authors declare that they have no competing interests.

## Authors' contributions

BX conceived the study, analyzed the data, and drafted the manuscript; SY researched and coordinated with experts in translating patients' postal codes into travel distances between patients' residences and the two hospital sites, and contributed substantially to the revision of the manuscript. BX takes responsibility for the paper as a whole.

## Consent

The patient consent requirement was waived by the Research Ethics Board for this study, as only de-identified data were used and it was not practical to obtain patient consent for such a retrospective study.

## References

[B1] Willcox S Seddon M Dunn S Edwards RT Pearse J Tu JV Measuring and reducing waiting times: A cross-national comparison of strategies Health Aff (Millwood) 2007 26 4 1078 87 10.1377/hlthaff.26.4.1078 17630450

[B2] Carroll RJ Horn SD Soderfeldt B James BC Malmberg L International comparison of waiting times for selected cardiovascular procedures J Am Coll Cardiol 1995 25 3 557 63 10.1016/0735-1097(94)00442-S 7860896

[B3] De Coster C Non-clinical factors associated with variation in cataract surgery waiting times in manitoba Can J Aging 2005 24 Suppl 1 Spring 47 58 16080136 10.1353/cja.2005.0043

[B4] Thind A Waiting time and equity in southwestern Ontario Department of Epidemiology and Biostatistics Seminar Presentation 2009

[B5] Ontario Ministry of Health and Long Term Care Ontario wait times strategy: Introduction 2011 http://www.health.gov.on.ca/transformation/wait_times/public/wt_public_mn.html

[B6] The Hospital of Central Connecticut ED wait times 2011 http://www.thocc.org/services/emergency/wait_times.aspx

[B7] Kevin MD Use iPhone apps for emergency room wait times with caution 2011 http://www.kevinmd.com/blog/2010/03/iphone-apps-emergency-room-wait-times-caution.html

[B8] London Health Science Centre Emergency department wait times 2011 http://www.lhsc.on.ca/About_Us/Accountability/Caring_for_our_Patients/Wait_Times/ED/

[B9] Dawson D Jacobs R Martin S Smith P Is patient choice an effective mechanism to reduce waiting times? Appl Health Econ Health Policy 2004 3 4 195 203 10.2165/00148365-200403040-00003 15901194

[B10] Dawson D Gravelle H Jacobs R Martin S Smith PC The effects of expanding patient choice of provider on waiting times: Evidence from a policy experiment Health Econ 2007 16 2 113 28 10.1002/hec.1146 16888753

[B11] Ryan M McIntosh E Dean T Old P Trade-offs between location and waiting times in the provision of health care: The case of elective surgery on the isle of wight J Public Health Med 2000 22 2 202 10 10.1093/pubmed/22.2.202 10912560

[B12] Howell GP Richardson D Forester A Sibson J Ryan JM Morgans BT Long distance travel for routine elective surgery: Questionnaire survey of patients' attitudes BMJ 1990 300 6733 1171 3 10.1136/bmj.300.6733.1171 2346803 PMC1662914

[B13] Conner-Spady B Sanmartin C Johnston G McGurran J Kehler M Noseworthy T Willingness of patients to change surgeons for a shorter waiting time for joint arthroplasty CMAJ 2008 179 4 327 32 18695180 10.1503/cmaj.071659 PMC2492973

[B14] Birk HO Henriksen LO Why do not all hip- and knee patients facing long waiting times accept re-referral to hospitals with short waiting time? questionnaire study Health Policy 2006 77 3 318 25 10.1016/j.healthpol.2005.08.002 16198018

[B15] Hoot NR Aronsky D Systematic review of emergency department crowding: Causes, effects, and solutions Ann Emerg Med 2008 52 2 126 36 10.1016/j.annemergmed.2008.03.014 18433933 PMC7340358

[B16] Moskop JC Sklar DP Geiderman JM Schears RM Bookman KJ Emergency department crowding, part 1--concept, causes, and moral consequences Ann Emerg Med 2009 53 5 605 11 10.1016/j.annemergmed.2008.09.019 19027193

[B17] Moskop JC Sklar DP Geiderman JM Schears RM Bookman KJ Emergency department crowding, part 2--barriers to reform and strategies to overcome them Ann Emerg Med 2009 53 5 612 7 10.1016/j.annemergmed.2008.09.024 19027194

[B18] Hoot NR Epstein SK Allen TL Jones SS Baumlin KM Chawla N Forecasting emergency department crowding: An external, multicenter evaluation Ann Emerg Med 2009 54 4 514,522.e19 10.1016/j.annemergmed.2009.06.006 PMC2800127 19716629

[B19] WESH.com New billboards display ER wait times 2011 http://www.wesh.com/health/20983530/detail.html# Available from:

[B20] Jones S Fox S Generations online in 2009 Pew Internet and American Life Project 2009 http://www.pewinternet.org/Reports/2009/Generations-Online-in-2009.aspx Accessed on ebruary 16 2011

[B21] Statistics Canada Postal code conversion file (PCCF), reference Guide 2007 Ottawa: Authority of the Minister responsible for Statistics Canada

[B22] Bow CJ Waters NM Faris PD Seidel JE Galbraith PD Knudtson ML Accuracy of city postal code coordinates as a proxy for location of residence Int J Health Geogr 2004 3 1 5 10.1186/1476-072X-3-5 15028120 PMC394341

[B23] White H Maximum likelihood estimation of misspecified models Econometrica 1982 50 1 25 10.2307/1912526

[B24] R Development Core Team R: A language and environment for statistical computing 2010 Vienna, Austria: R Foundation for Statistical Computing

[B25] Thompson DA Yarnold PR Williams DR Adams SL Effects of actual waiting time, perceived waiting time, information delivery, and expressive quality on patient satisfaction in the emergency department Ann Emerg Med 1996 28 6 657 65 10.1016/S0196-0644(96)70090-2 8953956

[B26] Pitrou I Lecourt AC Bailly L Brousse B Dauchet L Ladner J Waiting time and assessment of patient satisfaction in a large reference emergency department: A prospective cohort study, france Eur J Emerg Med 2009 16 4 177 82 10.1097/MEJ.0b013e32831016a6 19318959

[B27] Thompson DA Yarnold PR Williams DR Adams SL Effects of actual waiting time, perceived waiting time, information delivery, and expressive quality on patient satisfaction in the emergency department Ann Emerg Med 1996 28 6 657 65 10.1016/S0196-0644(96)70090-2 8953956

